# Supplementary material for: Controlled Nanostructuration of Cobalt Oxyhydroxide Electrode Material for Hybrid Supercapacitors
Source: Materials (Basel). 2021 Apr 29;14(9):2325. doi: 10.3390/ma14092325 (PMC8124577; doi:10.3390/ma14092325)
Supplement: Supplementary file 1 [file materials-14-02325-s001.zip › materials-1184894-supplementary.pdf]

materials

MDPI

Supplementary Information

Controlled Nanostructuration of Cobalt Oxyhydroxide Electrode Material for Hybrid Supercapacitors

Ronan Invernizzi [1,2], Liliane Guerlou-Demourgues [1,2,3], François Weill [1,2], Alexia Lemoine [4], Marie-Anne Dourges [5], Isabelle Baraille [4], Delphine Flahaut [2,4] and Jacob Olchowka [1,2,3,*]

1. CNRS, Univ. Bordeaux, Bordeaux INP, ICMCB UMR CNRS #5026, F-33600 Pessac, France; Ronan.INVERNIZZI@icmcb.cnrs.fr (R.I.); Liliane.Guerlou-Demourgues@enscbp.fr (L.G.-D.); Francois.Weill@icmcb.cnrs.fr (F.W.)
2. RS2E, Réseau Français sur le Stockage Electrochimique de l'Energie, FR CNRS #3459, CEDEX 1, F-80039 Amiens, France; dflahaut@univ-pau.fr
3. ALISTORE-ERI European Research Institute, FR CNRS #3104, CEDEX 1, F-80039 Amiens, France
4. CNRS/Univ. Pau & Pays Adour / E2S UPPA, Institut des Sciences Analytiques et de Physicochimie pour l'Environnement et les Matériaux − UMR 5254, F-64000 Pau, France; alexia.lemoine@univ-pau.fr (A.L.); isabelle.baraille@univ-pau.fr (I.B.)
5. Institut des Sciences Molaires, Univ. Bordeaux, UMR 5255, F-33405 Talence, France; marie-anne.dourges@u-bordeaux.fr

* Correspondence: jacob.olchowka@icmcb.cnrs.fr

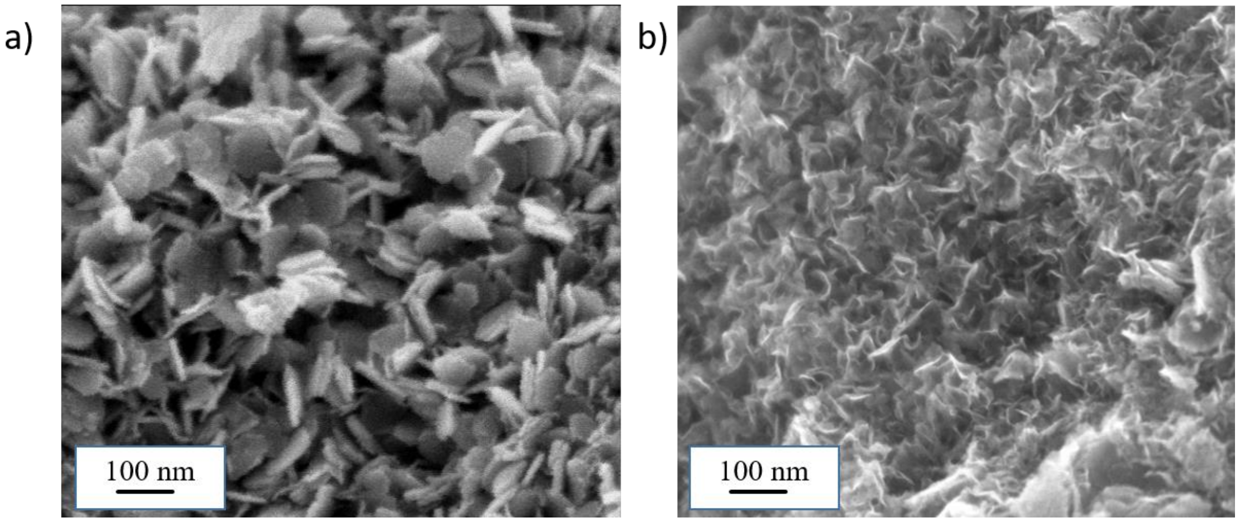

Figure S1. SEM images of a) β3-pH$_{14}$ and b) β3-pH$_{\nearrow}$. On these new images we can clearly seen the platelets like particles for β3-pH$_{14}$ whereas β3-pH$_{\nearrow}$ particles are smaller and more wavy.

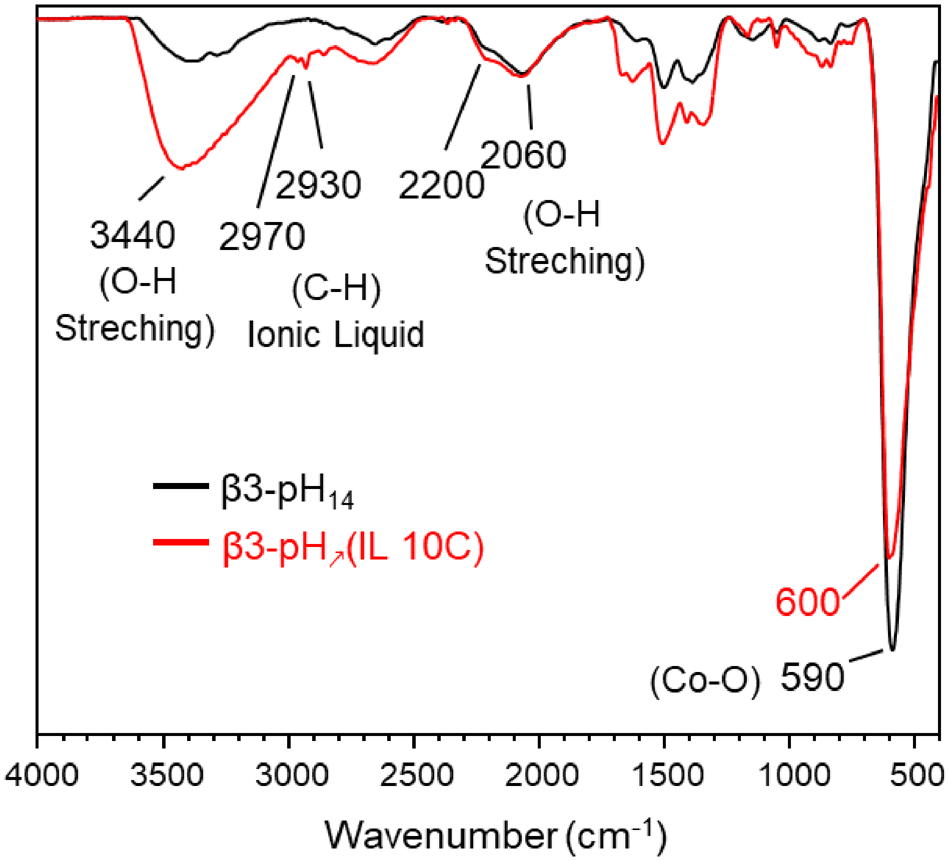

Figure S2. Infrared spectra of β3-pH_{14} (black curve) and β3-pH↗(IL 10C) (red curve). The bands at 2930 and 2970 cm^{-1} are attributed to ν_{C-H} bonds from the alkyl chain of imidazolium and confirm the presence of ionic liquid. Additionally, the more intense band with a maximum at 3440 cm^{-1} (attributed to O-H stretching of water) for β3-pH↗(IL 10C) compared to β3-pH_{14} can be linked to a higher amount of water (adsorbed or structural water) as expected for the elementary analysis.

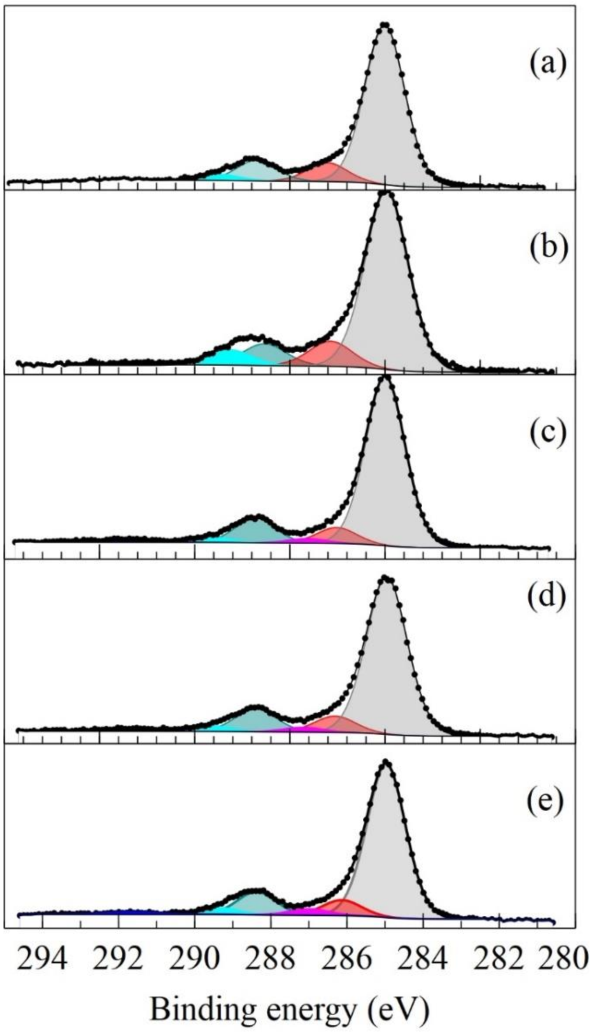

Figure S3. C 1s core peaks spectra a) β3-pH$_{14}$, b) β3-pH$_{\nearrow}$, c) β3-pH$_{\nearrow}$(IL 2C), d) β3-pH$_{\nearrow}$(IL 6C) and e) β3-pH$_{\nearrow}$(IL 10C).

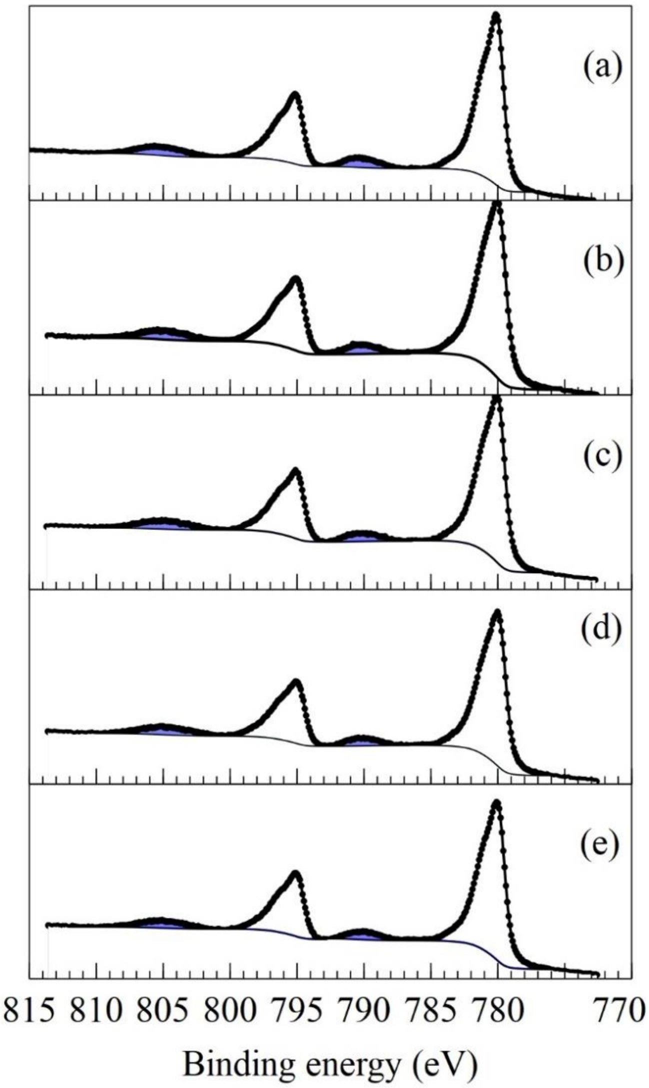

Figure S4. Co 2p core peaks spectra a) β3-pH14, b) β3-pH↗, c) β3-pH↗(IL 2C), d) β3-pH↗(IL 6C) and e) β3-pH↗(IL 10C).

The O 1s spectrum (not shown here) can be decomposed into three components at 529.5 eV, 531.2 eV and 533.3 eV. The component at 529.5 eV is assigned to the O^{2-} of oxide part of the material and the component at 531.2 eV to the hydroxyl groups of bulk and adsorbed species. The last component at 533.3 eV is resulting from the presence of water. The detection of Na in the sample is due to the presence of a few sodium atoms between the nanosheets and also to the synthesis conditions (see experimental part).

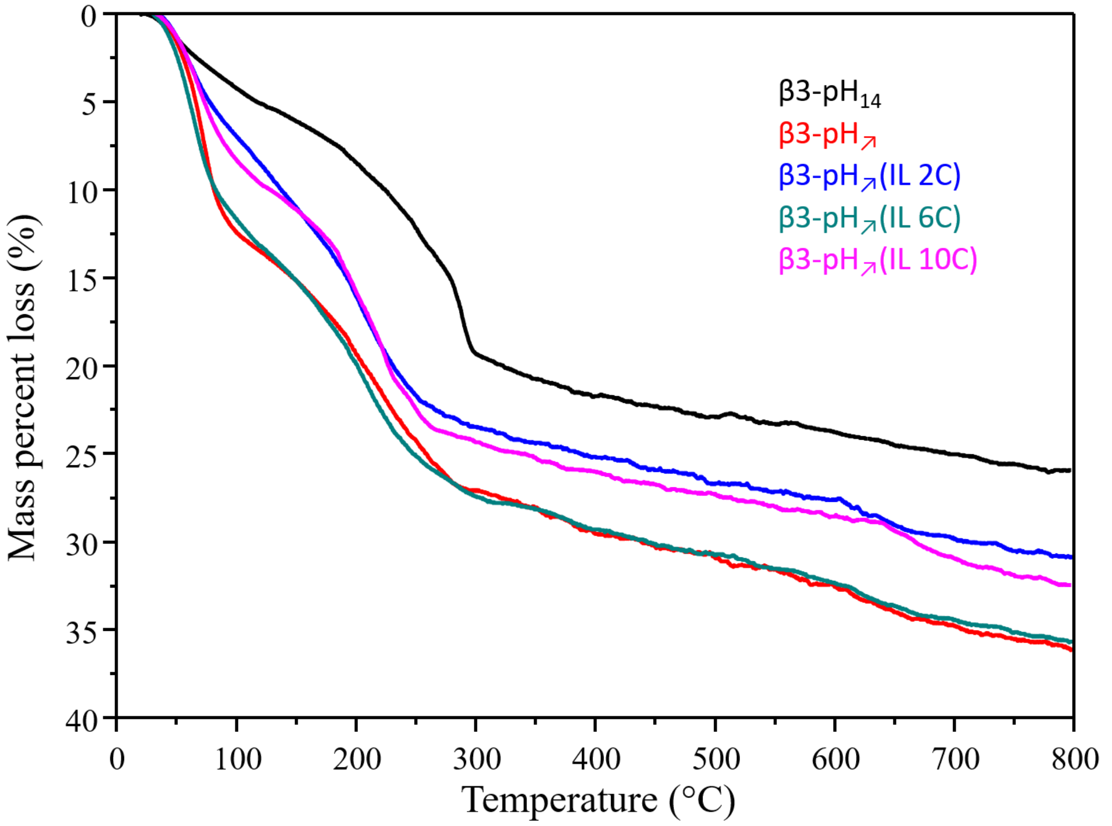

Figure S5. TGA data of β3-pH$_{14}$ (black curve), β3-pH↗ (red curve), β3-pH↗(IL 2C) (blue curve), β3-pH↗(IL 6C) (green curve) and β3-pH↗(IL 10C) (pink curve). All the compounds obtained by forward co-precipitation (β3-pH↗) exhibit higher weight losses between room temperature and 300 °C, which confirm a higher amount of water (adsorbed and interlayer water) as discussed in the manuscript.

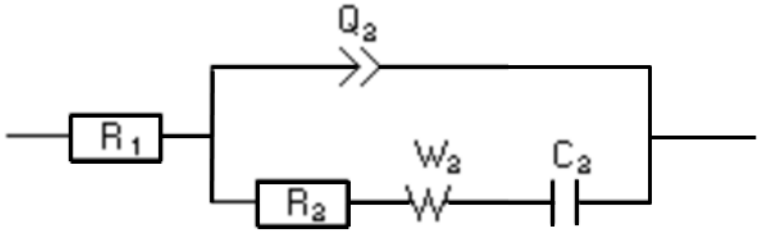

Figure S6. Equivalent circuit used to fit impedance spectroscopy data.

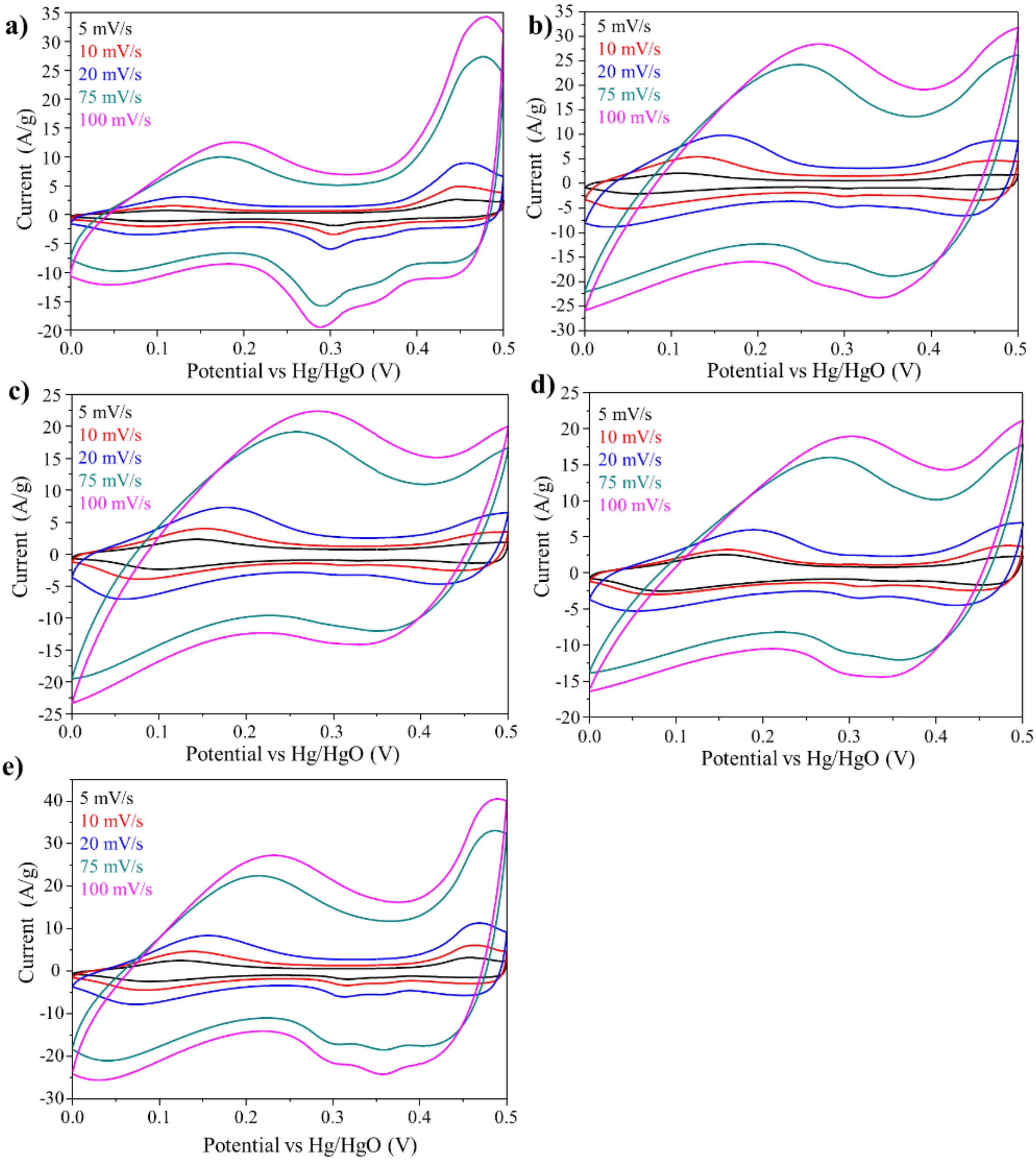

Figure S7. Cyclic Voltammetry of a) β3-pH$_{14}$, b) β3-pH↗, c) β3-pH↗(IL 2C), d) β3-pH↗(IL 6C) and e) β3-pH↗(IL 10C) performed at different scan rates in 5M-KOH electrolyte.

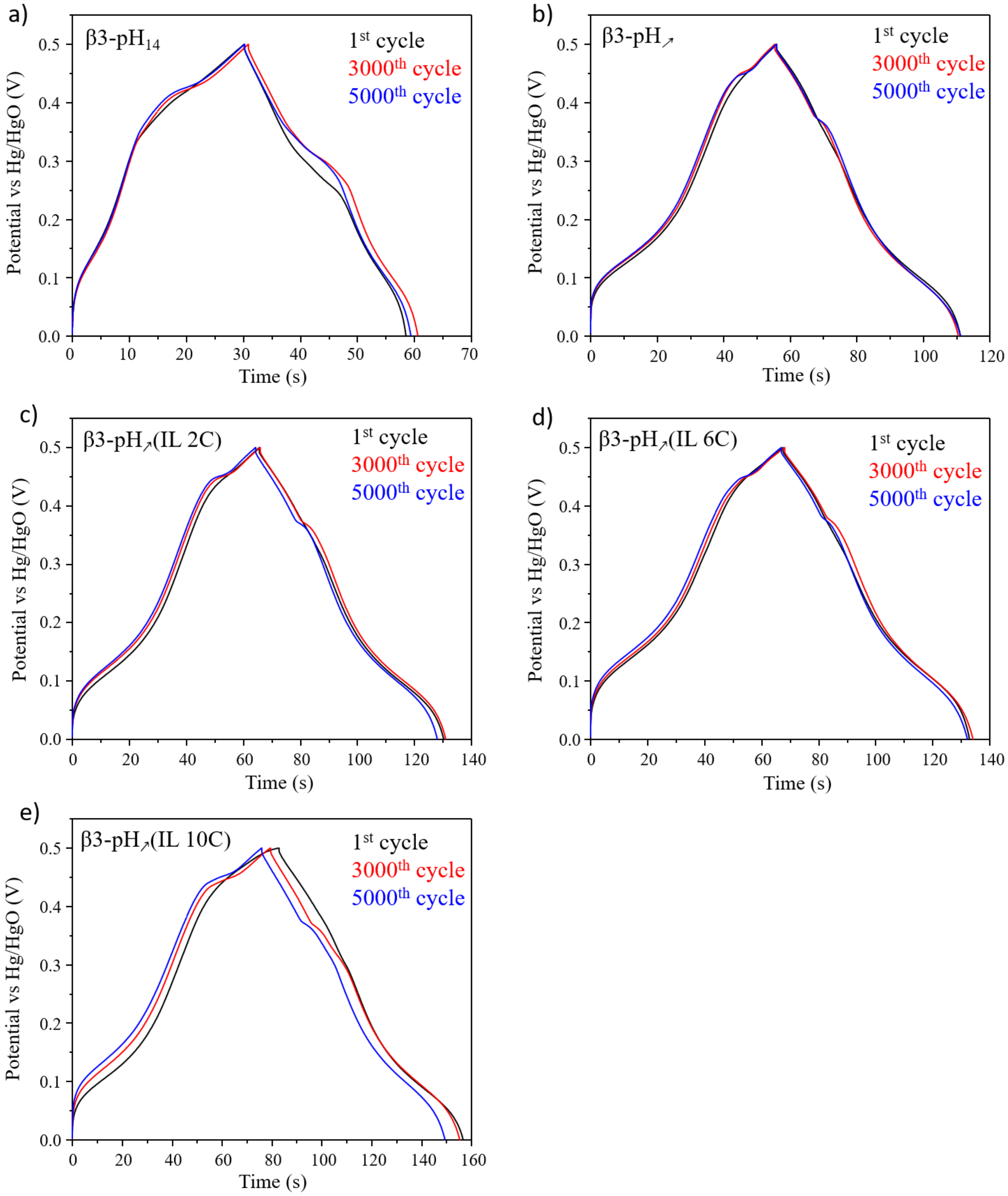

Figure S8. 1st, 3000th and 5000th galvanostatic charge/discharged curves of a) β3-pH14 b) β3-pH↗ c) β3-pH↗(IL 2C), d) β3-pH↗(IL 6C) and e) β3-pH↗(IL 10C) performed at 2 A/g in 5M-KOH electrolyte. For all electrodes materials it can be seen that the profile of the curve remains similar between the 1st and the 5000th cycle, which traduces a good structural stability upon long-term cycling.
